# Editorial: Harnessing single-cell insights: pioneering predictive markers for immunotherapy efficacy in solid tumors

**DOI:** 10.3389/fimmu.2025.1625920

**Published:** 2025-05-19

**Authors:** Zhongbao Zhou

**Affiliations:** Department of Urology, Beijing TianTan Hospital, Capital Medical University, Beijing, China

**Keywords:** single-cell RNA sequencing, biomarkers, immunotherapy, solid tumors, prognosis

The area of tumor immunotherapy has made great advances, owing principally to the development of immune checkpoint inhibitors, which constituted a breakthrough (1). However, developing predictive biomarkers capable of accurately forecasting individual response to immunotherapy remained a substantial issue. Despite improvements, we still don’t fully understand why some patients respond well while others don’t. The latest developments in single-cell analysis have transformed the area of immunotherapy. The technology’s ability to examine single immune cells inside the tumor microenvironment and immune system has enabled researchers to dive into hitherto unknown complexities (2). This Research Topic aims to tackle the issues of predictive biomarkers by harnessing the novel potential of single-cell research and technology. With investigating the actions and interactions of single immune cell, this Research Topic hope to acquire vital insights into the causes for diverse patients’ reactions to immunotherapy. These advances in single-cell analysis not only enrich the comprehension of immunotherapy, but they also have a chance to provide the door to tailored therapy, hence enhancing the results of immunotherapy treatments. The purpose of this project is to employ single-cell analysis to investigate prognostic variables for immunotherapy achievement throughout many types of solid tumors, offering knowledge regarding the challenges and possibilities given by each cancer type.

Impressively, we have carefully selected nine publications from many papers for this Research Topic. Seven of these studies revealed the role of single cell technology in recognizing molecular markers in different tumors, while the other two reviews summarized the role of single cell technology in melanoma and pancreatic cancer. We have summarized these studies below:

Antibody-dependent cellular phagocytosis (ADCP) immunotherapy is thought to be the new engine for precision therapy (3). Zhang et al. aimed to create an ADCP-based liver hepatocellular carcinoma (LIHC) risk stratification system and identify potential targets. They used a mix of single-cell RNA sequencing (scRNA-seq) (GSE149614) and bulk RNA-seq data to screen for ADCP modifying factors in LIHC and identified GYPA, CLDN18, and IRX5 as potential major target genes for ADCP regulation in LIHC. Validation using tissue and cell samples revealed that GYPA and CLDN18 were elevated in liver cancer tissues and cells. Additionally, *in vitro* suppression of CLDN18 reduced the malignant capability of liver cancer cells, indicating CLDN18 as an important ADCP regulatory receptor in LIHC.

Y-box-binding proteins (YBX) have a multifunctional role in tumor growth, metastasis, drug resistance via regulating transcription and translation pathways (4). Yuan et al. investigated the clinical characteristics expression, prognostic value, mutations, and methylation patterns of three genes from the YBX family (YBX1, YBX2, and YBX3) in 28 different kinds of cancer, and used the ssGSEA method to establish a novel YBXs score. The YBXs score has proven to be an effective predictor for the efficacy of a variety of cancer therapies, including immunotherapy. Furthermore, YBX2 was identified as a possible therapeutic target, which has considerably improved hepatocellular carcinoma (HCC) diagnostic and therapy options.

Glycosylation is critical for cell communication, immunological response, and protein stability (5). Jiang et al. obtained scRNA-seq data and transcriptome data from GSE197177 and GSE224411, respectively, to investigate the role of glycosylation mechanisms in pancreatic ductal adenocarcinoma (PDAC) development. They discovered that MGAT1 plays a crucial role in PDAC by controlling glycosylation levels in macrophages, which influences tumor growth and improves prognosis.

Hyaluronan mediated motility receptor (HMMR) is a protein-coding gene on chromosome that is expressed during cell cycle, peaking between the late G2 phase and early mitosis (6). However, its significance in HCC is unknown. Su et al. discovered that HMMR had good diagnostic performance, and HMMR knockdown inhibited the proliferation and migration of HCC cells *in vitro* using single-cell data analysis (GSE149614) and experimental verification. Additionally, HMMR was related with a bad prognosis in HCC patients, and re-staging using recursive partitioning analysis (RPA) provided an excellent prognosis predictive value, which might guide chemotherapy and targeted treatment.

The relationship between glycosylation and head and neck squamous cell carcinoma (HNSCC) is not well understood. Ma et al. identified key genes controlling glycosylation using scRNA-seq analysis, created a three-gene signature (SMS, HEG1 and MYO1B) that may accurately predict the prognosis of HNSCC and their response to immunotherapeutic therapies.

The tumor microenvironment (TME) is critical in the development and spread of lung adenocarcinoma (LUAD). Zhang et al. defined a comprehensive profile of immune cells in the LUAD microenvironment, including CD8+ T cells, CD4+ T cells, and myeloid cells, and developed a predictive model for LUAD based on exhausted CD8+ T cells marker genes (GALNT2, MTHFD1, FAM207A, KRT81, ORMDL3, IKZF3). The development of a LUAD single-cell atlas in this work provided novel insights into the tumor microenvironment and immune cell connections, emphasizing the significance of critical genes linked with exhausted CD8+ T cells. These results have enabled the construction of an efficient predictive model for LUAD and identified GALNT2 as a possible therapeutic target, therefore dramatically improving LUAD diagnostic and treatment options.

Melanoma, a malignant skin cancer caused by melanocytes, spreads rapidly and has a high death rate, particularly in the late stages. Melanoma cells use a variety of immune escape strategies, including immune recognition deficiencies and epithelial-mesenchymal transition (EMT), all of which have an influence on the effectiveness of therapy (7). Single-cell analysis methods have transformed our understanding of cancer heterogeneity and immune microenvironment interactions. He et al. gave an in-depth review of the use of single-cell methods for melanoma, stating that single-cell analysis keeps an effective method for clarifying the processes underlying reaction to therapy and resistance, ultimately resulting in the advancement of personalized melanoma treatments and enhanced outcomes for patients.

Pancreatic cancer is one of the deadliest cancers, with conventional therapeutic approaches having limited achievement. Recent advances in immune therapy have provided new optimism, but the specific TME of pancreatic cancer presents substantial difficulties to its successful implementation. Wang et al. investigated the revolutionary effect of single-cell technology on the comprehending and therapy of pancreatic cancer, as well as the importance of single-cell technology in separating the complex immune landscape of pancreatic cancer, emphasizing the finding of exhausted T cell profiles and polarization of macrophages states affecting response to therapy. They also discussed the obstacles and opportunities for converting these single-cell-based findings into clinical application, highlighting the importance of collaborative research and the use of artificial intelligence to overcome present limits.

The STING (Stimulator of Interferon Genes) pathway serves as a key component of the innate immune system, recognizing both internal and external DNA damage and initiating protective immune responses (8). This signaling cascade is particularly important in the context of cancer biology, where it influences the body’s ability to detect and respond to malignant cells. Ding et al. revealed the role of STING related pathways in LUAD by using scRNA-seq technology. They developed a predictive model based on the STING pathway and confirmed its therapeutic potential in regulating immune response by knocking down the key gene ERRFI1 in STING pathway.

All in all, scRNA-seq technology holds great promise for advancing cancer immunotherapy. By enabling high-resolution analysis of the tumor microenvironment, it allows for the identification of diverse immune cell populations and their functional states. This technology facilitates a deeper understanding of tumor-immune interactions, guiding the development of more precise and personalized immunotherapeutic strategies. As single-cell approaches continue to evolve, they are expected to uncover novel biomarkers and therapeutic targets, ultimately improving treatment efficacy and patient outcomes in cancer immunotherapy.

## References

[1] Liu Z Zhu Y Xie H Zou Z. Immune-mediated hepatitis induced by immune checkpoint inhibitors: Current updates and future perspectives. Front Pharmacol. (2023) 13:1077468. doi: 10.3389/fphar.2022.1077468 36699050 PMC9868416

[2] Tirosh I Suva ML. Cancer cell states: Lessons from ten years of single-cell RNA-sequencing of human tumors. Cancer Cell. (2024) 42:1497–506. doi: 10.1016/j.ccell.2024.08.005 39214095

[3] Van Wagoner CM Rivera-Escalera F Jaimes-Delgadillo NC Chu CC Zent CS Elliott MR. Antibody-mediated phagocytosis in cancer immunotherapy. Immunol Rev. (2023) 319:128–41. doi: 10.1111/imr.13265 PMC10615698 37602915

[4] Guens GP. YB-1 protein in breast cancer (Scientific and personal meetings with professor ovchinnikov). Biochem (Mosc). (2022) 87:S86–47. doi: 10.1134/S0006297922140073 35501988

[5] Sun R Kim AMJ Lim SO. Glycosylation of immune receptors in cancer. Cells. (2021) 10:1100. doi: 10.3390/cells10051100 34064396 PMC8147841

[6] He Z Mei L Connell M Maxwell CA. Hyaluronan mediated motility receptor (HMMR) encodes an evolutionarily conserved homeostasis, mitosis, and meiosis regulator rather than a hyaluronan receptor. Cells. (2020) 9:819. doi: 10.3390/cells9040819 32231069 PMC7226759

[7] Pedri D Karras P Landeloos E Marine JC Rambow F. Epithelial-to-mesenchymal-like transition events in melanoma. FEBS J. (2022) 289:1352–68. doi: 10.1111/febs.16021 33999497

[8] Samson N Ablasser A. The cGAS-STING pathway and cancer. Nat Cancer. (2022) 3:1452–63. doi: 10.1038/s43018-022-00468-w 36510011

